# Naturally menstruating women exhibit lower cardiovagal baroreflex sensitivity than oral contraceptive users during the lower hormone phase

**DOI:** 10.1113/EP091394

**Published:** 2023-10-25

**Authors:** Sara E. Mascone, Dain W. Jacob, Lauren E. Eagan, Jennifer L. Harper, Jacqueline K. Limberg, Sushant M. Ranadive

**Affiliations:** ^1^ Department of Kinesiology, School of Public Health University of Maryland College Park Maryland USA; ^2^ Department of Nutrition and Exercise Physiology University of Missouri Columbia Missouri USA

**Keywords:** baroreflex sensitivity, blood pressure regulation, sex hormones

## Abstract

The present study evaluated cardiovagal baroreflex sensitivity (BRS) across the menstrual/pill cycle in naturally menstruating women (NAT women) and women using oral hormonal contraceptives (OCP women). In 21 NAT women (23 ± 4 years old) and 22 OCP women (23 ± 3 years old), cardiovagal BRS and circulating concentrations of estradiol and progesterone were evaluated during the lower hormone (early follicular/placebo pill) and higher hormone (late follicular to early luteal/active pill) phases. During the lower hormone phase, cardiovagal BRS up, down and mean gain were lower in NAT women (15.6 ± 8.3, 15.2 ± 6.1 and 15.1 ± 7.1 ms/mmHg) compared with OCP women (24.7 ± 9.4, 22.9 ± 8.0 and 23.0 ± 8.0 ms/mmHg) (*P* = 0.003, *P* = 0.002 and *P* = 0.003, respectively), and higher oestrogen (*R* ^2^ = 0.15, *P* = 0.024), but not progesterone (*R* ^2^ = 0.06, *P* = 0.18), concentrations were predictive of lower BRS mean gain. During the higher hormone phase, higher progesterone concentrations were predictive of lower BRS mean gain (*R* ^2^ = 0.12, *P* = 0.024). A multivariate regression model revealed group (NAT or OCP) to be a significant predictor of cardiovagal BRS mean gain in the lower hormone phase when hormone concentrations were adjusted for (*R* ^2^ = 0.36, *P* = 0.0044). The multivariate regression model was not significant during the higher hormone phase (*P* > 0.05). In summary, cardiovagal BRS is lower in NAT compared with OCP women during the lower hormone phase of the menstrual/pill cycle and might be associated with higher oestrogen concentrations. In contrast, during the higher hormone phase of the menstrual/OCP cycle, higher progesterone concentrations were predictive of lower cardiovagal BRS.

## Abstract (highlights)

- **What is the central question of this study?**
  Does cardiovagal baroreflex sensitivity (BRS) differ between naturally menstruating women (NAT women) and women using oral contraceptives (OCP women)?
- **What is the main finding and its importance?**
  The main findings are as follows: (1) NAT women exhibit lower cardiovagal BRS than OCP women during the lower hormone phase of the menstrual or pill cycle; and (2) circulating oestrogen concentrations are significant predictors of cardiovagal BRS during the lower hormone phase, with higher oestrogen concentrations predicting lower BRS. The present data advance our understanding of the effect of endogenous ovarian hormones and OCP use on cardiovascular control mechanisms.

## 1 INTRODUCTION

Blood pressure is a highly regulated variable, and numerous cardiovascular control mechanisms are in place in healthy adults to maintain blood pressure within a homeostatic range. Failure of one or more of these mechanisms leads to heightened blood pressure and eventual dysfunction in cardiovascular control machinery, as is seen with impaired blood pressure regulation with ageing (Barnes et al., 2012). One such cardiovascular control mechanism is the cardiovagal baroreflex. Baroreflex sensitivity (BRS) characterizes the efficiency of the baroreflex in regulating arterial blood pressure and heart rate. For instance, lower cardiovagal BRS is associated with higher blood pressure in older adults, with a stronger association present in women than in men (Zhou et al., 2022). Furthermore, cardiovagal BRS is lower in post‐ compared with premenopausal women, suggesting a prominent role of age and/or ovarian hormones in cardiovagal BRS (Barnes et al., 2012).

The effect of ovarian hormones (oestrogens and progesterone) on cardiovagal BRS is debatable, with some studies showing no differences in cardiovagal BRS across the menstrual cycle (Hayashi et al., 2006; Minson et al., 2000a; Vollebregt et al., 2006), whereas other data suggest higher cardiovagal BRS during the late follicular (pre‐ovulation) compared with the early follicular phase of the menstrual cycle (Tanaka et al., 2003). Even less clear are the potentially differential effects of endogenous [natural menstrual cycles (NAT)] compared with exogenous [oral hormonal contraceptive pill (OCP)] ovarian hormones on baroreflex control. In this context, OCP women have exhibited greater cardiovagal BRS during the lower hormone (placebo pill) phase compared with the higher hormone (active pill) phase (Minson et al., 2000b). However, when OCP and NAT women were compared at a single point in the menstrual cycle (lower hormone phase), cardiovagal BRS was similar between NAT and OCP women (Wilczak et al., 2013). Notably, endogenous oestrogens are likely to drive the cardioprotection experienced in premenopausal women (Colafella & Denton, 2018; Stanhewicz et al., 2018). Unfortunately, cardiovagal BRS during higher hormone phases has not yet been compared between NAT and OCP women, and ovarian hormone concentrations have not been considered in previous studies with NAT and/or OCP women. Thus, it is unclear whether endogenous ovarian hormone concentrations (i.e., oestrogens, progesterone) and/or OCP use differentially influence cardiovagal BRS, highlighting two important gaps in knowledge.

The purpose of the present study was to investigate the influence of circulating oestrogen (17β‐estradiol) and progesterone on cardiovagal BRS during the lower hormone (early follicular/placebo pill) and higher hormone (late follicular to early luteal/active pill) phase in NAT and OCP women. We hypothesized that positive associations exist between endogenous circulating oestrogen and progesterone concentrations and BRS. In accordance with previous work, we also hypothesized that BRS would be higher during the lower hormone versus the higher hormone phase of the pill cycle for OCP women. Last, we hypothesized that women using OCPs would exhibit similar cardiovagal BRS when compared with NAT women, although group differences might appear during the higher hormone phases when oestrogen concentrations are highest.

## 2 MATERIALS AND METHODS

### 2.1 Ethical approval

All study protocols were approved by the Institutional Review Boards (IRBs) at the University of Maryland, College Park (UMD; IRB #1290733) and the University of Missouri (MU; IRB #2011312, NCT03606434). All protocols conformed to standards set by the *Declaration of Helsinki* (excluding registration in a database), and all participants submitted written informed consent after trained personnel explained all study protocols.

### 2.2 Participants

Forty‐three young, healthy women who were NAT (*n* = 21) or taking OCPs (*n* = 22) participated in the study. Participants were pooled from previously published studies conducted at UMD (Eagan et al., 2022, 2021) and MU (Jacob et al., 2021, 2022) to test new hypotheses proposed herein. Participants had no history of cardiovascular disease or other diseases (pulmonary conditions, metabolic conditions, cancer or stroke) that might alter vascular function, were not taking medications apart from oral contraceptives (antibiotics, non‐steroidal anti‐inflammatory drugs and blood pressure medication). Participants were not pregnant, had a body mass index of <30 kg/m^2^ and a blood pressure of <140/90 mmHg. Additionally, all women confirmed regular menstrual cycles (Munro et al., 2018) or regular and consistent use of the same brand and formulation of oral contraceptives for the last 6 months. All women also confirmed that they did not currently have an intra‐uterine device or other hormonal implant. See Table 1 for the type of OCPs and mean and range of OCP formulations included in the present study.

**TABLE 1:** Participant characteristics, with oral contraceptive pill information for participants in the OCP group.

OCP details	Characteristic	All	OCP	NAT
	Count, *n*	43	22	21
	Age, years	23 (4)	23 (3)	23 (4)
	Height, m	1.64 (0.04)	1.64 (0.05)	1.65 (0.04)
	Weight, kg	61.6 (8.1)	59.8 (8)	63.5 (7.6)
	BMI, kg/m^2^	22.7 (2.7)	22.1 (2.7)	23.4 (2.5)
OCP type	Monophasic	–	19	–
	Biphasic	–	2	–
	Triphasic	–	1	–
OCP formulation	Estradiol dosage, mg	–	0.026 (0.007) Range: 0.020–0.035	–
	Progestin dosage, mg	–	0.50 (0.66) Range: 0.10–3.00	–
LH	SBP, mmHg	111 (9)	113 (10)	109 (8)
	DBP, mmHg	69 (6)	69 (5)	69 (6)
	HR, beats/min	62 (9)	61 (10)	63 (9)
HH	SBP, mmHg	111 (8)	112 (10)	109 (6)
	DBP, mmHg	67 (6)	67 (5)	68 (6)
	HR, beats/min	65 (10)	64 (11)	65 (9)

*Note*: Data are presented as means (SD). All between‐group participant characteristic comparisons are *P* > 0.05. Abbreviations: BMI, body mass index; DBP, diastolic blood pressure; HH, high hormone; HR, heart rate; LH, low hormone; NAT, naturally menstruating women; OCP, oral contraceptive pill using women; SBP, systolic blood pressure.

All participants came to the laboratory during two phases of the menstrual cycle or oral contraceptive pill pack [the lower hormone (LH) and higher hormone (HH) phases], completed in random order of convenience. The lower hormone phase visits were completed during the early follicular phase (within 6 days of menses onset) or the placebo pill phase (within the 7 days of placebo pills), and the higher hormone phase visits were completed during the late follicular to early luteal phase (between 1 and 4 days post‐ovulation) or active pill phase (during the highest exogenous oestrogen and progesterone concentrations). To schedule the higher hormone phase visits, NAT women were asked to notify researchers immediately when ovulation was indicated by a urine‐based ovulation kit (Clearblue Advanced Digital Ovulation Test, Swiss Precision Diagnostics, Geneva, Switzerland).

### 2.3 Continuous monitoring: Beat‐to‐beat blood pressure and ECG

Throughout the entirety of testing, participants remained supine in the temperature‐controlled, dimly lit laboratory spaces. Participants were outfitted with a beat‐to‐beat finger blood pressure (BP) cuff on their right‐hand middle, index or ring finger for finger photoplethysmography and five electrodes for four‐lead ECG (UMD) or three electrodes for a three‐lead ECG (UM). Participants were asked to remain still and quiet for the resting ECG and finger BP baseline. At least 5 min of resting ECG and BP data were collected in LabChart (v.8.0; ADInstruments, Bella Vista, NSW, Australia). Owing to the nature of the present retrospective analysis, MU participants were outfitted with a breathing mask connected to room air.

### 2.4 Cardiovagal BRS

Cardiovagal BRS was measured as the change in R–R interval (RRI; the time between two sequential R waves from the ECG) over the per unit change in systolic BP (SBP, in milliseconds per millimetre of mercury) (Swenne, 2013). Cardiovagal BRS up, down and mean gain were extracted from the resting ECG RRI and SBP data. At least 5 min of continuous, high‐quality data collected at 1000 Hz during quiet supine rest were collected for BRS analysis using the sequence method as part of a semi‐automated program (Cardioseries v.2.7; Sao Paulo, Brazil) used by a single investigator (S.E.M.). Sequences of at least three consecutive beats with either increases in SBP (SBP threshold = 1.0 mmHg) accompanied by lengthening of the RRIs (up sequence, RRI threshold = 5 ms) or decreases in SBP accompanied by shortening of the RRIs (down sequence) with a correlation of ≥0.85 were used. Cardiovagal BRS up, down and mean gain are the combined slope of the linear regression between RRI and SBP for up, down or all sequences combined, respectively, and values are reported in milliseconds per millimetre of mercury.

### 2.5 Endogenous ovarian hormone concentrations

At UMD and MU, quantification of circulating serum concentrations of the ovarian hormones oestrogen and progesterone were conducted as previously described (Eagan et al., 2021; Jacob et al., 2022). At UMD and MU, fasted venous blood samples were collected into serum separator tubes (UMD) or red top tubes (UM) that sat at room temperature for 45 min before being centrifuged at 1500*g* for 15 min (UMD) or for 15 min before being centrifuged at 1900*g* for 10 min (MU), all at 4°C. Serum was aliquoted and stored at −80°C until future analysis of oestrogen and progesterone concentrations (UMD) or stored at 4°C before being sent for external quantification (MU, Quest Diagnostics). At UMD, enzyme‐linked immunosorbent assays were used to quantify endogenous 17β‐estradiol (10 pg/mL sensitivity) and progesterone (0.1 ng/mL sensitivity), according to the manufacturer's instructions (ALPCO, Salem, NH, USA). Absorbances were measured at 450 nm and duplicate sample values averaged.

### 2.6 Statistical analyses

Data were assessed for normality using Shapiro–Wilks tests. Three outliers were identified via Tukey's method (one each from lower hormone up gain, lower hormone mean gain and higher hormone down gain) and removed from BRS up, down and mean gain data. Any participants who did not have at least three up and down sequences were not included in BRS analysis; the number of participants included in each variable is reported within table and figure legends. Student's unpaired *t*‐tests were performed to compare participant characteristics (age, height, weight and body mass index) of NAT and OCP women, and Student's paired *t*‐tests were performed to compare participant characteristics between lower and higher hormone phases. Student's unpaired *t*‐tests were performed to compare ovarian hormone concentrations between NAT and OCP women, and Student's paired *t*‐tests were performed to compare ovarian hormone concentrations between lower and higher hormone phases. Two‐way repeated‐measures ANOVAs for up, down and mean gain, with factors of phase (lower or higher hormone) and group (NAT or OCP status), were performed, and Student's unpaired or paired *t*‐tests were used *post hoc* to investigate any significant model effects or interactions. Univariate simple linear regressions were performed to investigate whether ovarian hormone concentrations could significantly predict BRS mean gain. Separate univariate simple linear regressions were performed for the lower and higher hormone phases and for oestrogen and progesterone concentrations. Lastly, a multiple linear regression model was built to assess whether NAT/OCP status or ovarian hormones were significant predictors of BRS mean gain. Given that BRS mean gain is the summation of the slopes of all up and down sequences, only BRS mean gain was used as an outcome for all regressions. The value of α was set a priori to 0.05 for all statistical tests, and all data are presented as means ± SD. All statistical analyses were performed via SPSS v.27 (IBM, Chicago, IL, USA), and graphs were created in GraphPad Prism v.9 (GraphPad, San Diego, CA, USA).

## 3 RESULTS

### 3.1 Participant characteristics and ovarian hormone concentrations

Baseline participant characteristics and OCP type and formulations are reported in Table 1. Age, height, weight and body mass index were not different between groups (*P* > 0.05 for all). Resting SBP, diastolic blood pressure (DBP) and heart rate were not different between phases and between groups (*P* > 0.05), suggesting that participants were well matched. About 86% of OCPs used were monophasic, with estradiol and progestin average dosages of 0.026 ± 0.007 and 0.50 ± 0.66 mg, respectively. Table 2 contains ovarian hormone concentrations. The oestrogen and progesterone concentrations did not differ between NAT and OCP women in the lower hormone phase, whereas NAT women had greater endogenous oestrogen and progesterone concentrations during the higher hormone phase when compared with OCP (*P* < 0.0001 and *P* = 0.004, respectively).

**TABLE 2:** Ovarian hormone concentrations for OCP and NAT participants.

Hormone		All (*n* = 43)	OCP (*n* = 22)	NAT (*n* = 21)	OCP vs. NAT *P*‐value
LH	E2, pg/mL	53.22 (33.97)	45.24 (24.92)	61.65 (40.50)	0.14
	P4, ng/mL	0.90 (0.64)	0.83 (0.69)	0.98 (0.59)	0.47
HH	E2, pg/mL	88.40 (88.37)^*^	36.06 (27.53)	143.36 (97.15)^*^	**0.00002**
	P4, ng/mL	2.10 (2.60)^*^	0.99 (0.83)	3.24 (3.28)^*^	**0.004**

*Note*: Data are presented as means (SD). Significant *post hoc* Student's unpaired *t*‐tests between NAT and OCP P‐values are shown in bold. ^*^ *P* < 0.05 compared with the low hormone phase (*post hoc* Student's paired *t*‐tests). LH OCP, E2 and P4, *n* = 19; LH NAT, *n* = 18; HH OCP, *n* = 21; HH NAT, *n* = 20. Abbreviations: E2, oestrogen; HH, high hormone; LH, low hormone; NAT, naturally menstruating women; OCP, oral contraceptive pill using women; P4, progesterone.

### 3.2 Cardiovagal BRS: Up, down and mean gain

**FIGURE 1:**
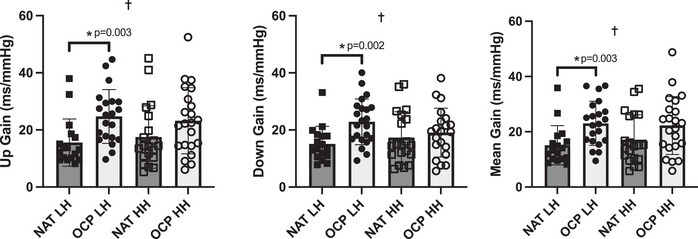
Up, down and mean gain baroreflex sensitivity in naturally menstruating (NAT) women (squares) or women taking an oral contraceptive (OCP; circles) during the low hormone phase (LH; filled symbols) or high hormone phase (HH; open symbols). There were no significant effects of phase or interactions; however, † denotes significant effects of group (NAT or OCP; *P* = 0.006, *P* = 0.04 and *P* = 0.008 for up, down and mean gain, respectively). ^*^ *P* < 0.05 between NAT and OCP women. NAT LH, *n* = 17; OCP LH, *n* = 21 for up and mean gain and *n* = 22 for down gain; NAT HH, *n* = 20; OCP HH, *n* = 21 for down gain and *n* = 22 for up and mean gain.

There was a significant main effect of group (NAT or OCP) for up, down and mean gain (*P* = 0.0055, *P* = 0.037 and *P* = 0.0083, respectively), despite no effects of phase (low or high hormone) and no main interaction effect (*P* = 0.057) between group and phase for cardiovagal BRS up, down or mean gain (*P* > 0.05 for all). Specifically, NAT women exhibited significantly lower up, down and mean gain than OCP women during the lower hormone phase (*P* = 0.003, *P* = 0.002 and *P* = 0.003, respectively; Figure 1). During the higher hormone phase, up, down and mean gain were not different between NAT and OCP women (*P* > 0.05 for all).

### 3.3 Ovarian hormone concentrations and cardiovagal BRS mean gain

**FIGURE 2:**
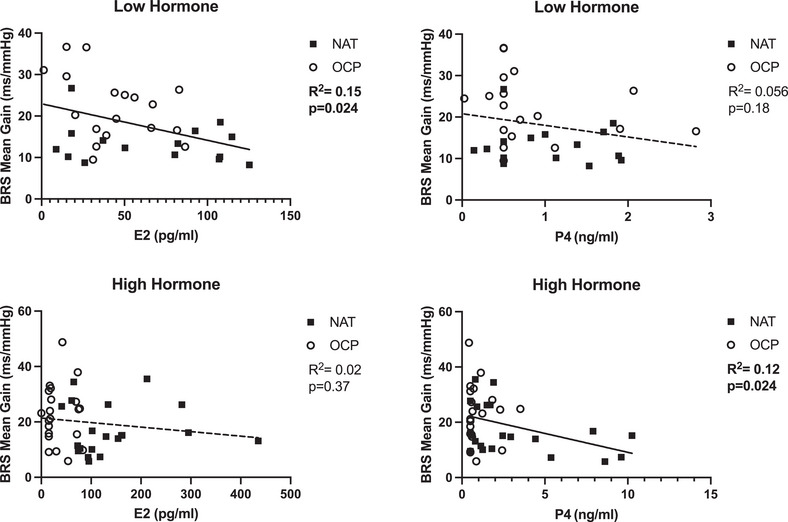
Univariate linear regressions of oestrogen concentrations (left column) or progesterone concentrations (right column) and mean gain for the low hormone phase (top graphs) or high hormone phase (bottom graphs). Significant relationships are denoted by a continuous line and bold *R* ^2^ and *P*‐values. Abbreviations: BRS, baroreflex sensitivity; E2, oestrogen; NAT, naturally menstruating; OCP, oral contraceptive pill; P4, progesterone.

Simple univariate linear regressions were performed for oestrogen concentrations, progesterone concentrations for group (NAT or OCP) and mean gain for the lower and higher hormone phases. In the lower hormone phase, oestrogen concentrations were inversely associated with mean gain, with higher oestrogen concentrations predicting lower mean gain (*R* ^2^ = 0.15, *P* = 0.024; Figure 2). However, during the higher hormone phase, oestrogen concentrations were not a significant predictor of mean gain (*R* ^2^ = 0.020, *P* = 0.37; Figure 2). Furthermore, progesterone concentrations were not a significant predictor of mean gain during the lower hormone phase (*R* ^2^ = 0.056, *P* = 0.18; Figure 2). However, during the higher hormone phase, progesterone concentrations were inversely associated with mean gain, with higher progesterone concentrations predicting lower mean gain (*R* ^2^ = 0.12, *P* = 0.0024; Figure 2; see Supplementary Figure 1 for linear regressions with low and high hormone phase oestrogen and progesterone concentrations combined).

To investigate these relationships further, a multiple linear regression model was built for the lower and higher hormone phases separately with mean gain as the outcome variable and group (NAT or OCP), oestrogen and progesterone concentrations as predictor variables. Table 3 contains β values, 95% confidence intervals, and *P*‐values for each variable in the model. For the lower hormone phase, the overall model was significant (*R* ^2^ = 0.36, *P* = 0.0044), with only group being a significant predictor of mean gain (*P* = 0.0050) after adjustment for ovarian hormone concentrations. The higher hormone phase model was not significant, and none of the predictors in the model were significant (*P* > 0.05 for all).

**TABLE 3:** Multivariate linear regression assessing group and sex hormones with baroreflex sensitivity mean gain in the low and high hormone phases.

Hormone phase and group		β (95% CI)	*P*‐value
LH	Intercept	17.06 (11.08, 23.04)	<0.0001
	Group	7.50 (2.46, 12.54)	0.0050
	E2	−0.053 (−0.15, −0.041)	0.26
	P4	−0.069 (−4.82, 4.68)	0.98
HH	Intercept	20.66 (11.37, 29.95)	<0.0001
	Group	3.06 (−5.528, 11.64)	0.47
	E2	0.00059 (−0.044, 0.046)	0.98
	P4	−1.11 (−2.454, 0.23)	0.10

*Note*: Data are presented as means (SD). LH OCP, E2 and P4 *n* = 19; LH NAT, *n* = 18; HH OCP, *n* = 21; HH NAT, *n* = 20. Abbreviations: E2, oestrogen; HH, high hormone; LH, low hormone; NAT, naturally menstruating women; OCP, oral contraceptive pill using women; P4, progesterone.

## 4 DISCUSSION

The main findings of the present study are threefold: (1) NAT women exhibit lower cardiovagal BRS than OCP women during the lower hormone phase of the menstrual or pill cycle; (2) circulating oestrogen (17β‐estradiol) concentrations are significant predictors of cardiovagal BRS during the lower hormone phase, with higher oestrogen concentrations predicting lower BRS; and (3) group (NAT or OCP) remains a significant predictor of BRS after adjustment for ovarian hormone concentrations. Together, these data advance our understanding of the effect of endogenous ovarian hormones and OCP use on cardiovascular control mechanisms.

### 4.1 Cardiovagal BRS across hormone phases

Elevated cardiovagal BRS denotes greater changes in heart rate per unit change in blood pressure. Based on previous literature suggesting an increase in cardiovagal BRS with the peak in oestrogens pre‐ovulation in NAT women (Tanaka et al., 2003), we hypothesized that cardiovagal BRS would increase when endogenous ovarian hormones are high. Contrary to our hypothesis, there was no difference in cardiovagal BRS between the lower and higher hormone phase in NAT women, and this is in line with work from others (Cooke et al., 2002; Hayashi et al., 2006; Minson et al., 2000a; Vollebregt et al., 2006). Discrepancies between studies might be attributable to the techniques used to measure BRS (i.e., spontaneous non‐invasive measurement, the use of lower‐body negative pressure or neck suction/pressure, or the modified Oxford technique) and the time point in the menstrual cycle when NAT women were assessed, because the present study did not include a pre‐ovulation late follicular phase.

Furthermore, previous data suggest pill phase‐dependent differences in cardiovagal BRS in OCP women (Minson et al., 2000b), with higher BRS during the lower (placebo) versus higher (active pill) hormone phase; however, the present study did not find this to be the case, because cardiovagal BRS did not differ across phases in the present cohort of OCP women. Remarkably, the present cohort of OCP women also exhibited cardiovagal BRS nearly twofold higher than those reported for OCP women by Minson et al. (2000b). Although OCP women were studied during similar lower and higher hormone phase day ranges in both studies, the disparity in BRS values between studies is observed only in OCP women, because the NAT women in the present study and the study by Minson et al. (2000a) exhibit numerically similar BRS values. The disparity might be attributable to participant age [∼30 years in the study by Minson et al. (2000a) compared with ∼23 years in the present study], OCP generation [>20 years between the study by Minson et al. (2000a) and the present study] or OCP formulation [between 0.030 and 0.035 mg ethinyl estradiol and low‐dose progestin in the study by Minson et al. (2000a) in comparison to between 0.020 and 0.035 mg ethinyl estradiol and 0.10–3.00 mg progestin in the present study]. For example, third‐generation OCPs have been associated with higher inflammatory and thrombotic cardiovascular risk profiles, suggesting that OCP generation might alter various cardiovascular mechanisms (Döring et al., 2004).

### 4.2 Cardiovagal BRS in NAT versus OCP women

A previous study found that cardiovagal BRS does not differ between NAT and OCP groups when compared during the lower hormone phase (days 4–8 of the menstrual or pill cycle) (Wilczak et al., 2013). In contrast, the present study found that OCP women exhibited greater cardiovagal BRS than NAT women during the lower hormone phase of the menstrual/pill cycle, but not during the higher hormone phase. To our knowledge, the present study is the first to compare cardiovagal BRS directly between NAT and OCP groups during the higher hormone phase. The contrasting findings between previous data and the present study of lower cardiovagal BRS in NAT women during the lower hormone phase might be attributable to differences in OCP generations or formulations, as previously mentioned. The differences could also be attributable to greater systemic inflammation. Indeed, greater inflammation has been associated with lower cardiovagal BRS in young and middle‐aged/older men (Babcock et al., 2022), and we have previously reported greater inflammation in NAT women compared with OCP women during the lower hormone, but not higher hormone phase (Eagan et al., 2021).

### 4.3 Cardiovagal BRS and oestrogen and progesterone concentrations

Aligning with our hypotheses, oestrogen (17β‐estradiol) concentrations were negatively associated with cardiovagal BRS during the lower hormone phase. The present association between greater oestrogen concentrations and lower cardiovagal BRS during the lower hormone phase in all NAT and OCP women might suggest a deleterious effect of higher oestrogen on cardiovagal BRS during the lower hormone phase. However, these findings are contrary to previously published data suggesting increased, rather than decreased, cardiovagal BRS when oestrogens peak during the late follicular phase (pre‐ovulation) in NAT women (Tanaka et al., 2003). To investigate the influence of sex hormones on cardiovagal BRS further, one study used a gonadotrophin‐releasing hormone antagonist, which blocks endogenous ovarian sex hormone production (Brunt et al., 2013). Surprisingly, following the blockade of endogenous ovarian sex hormone release with the gonadotrophin‐releasing hormone antagonist, oestrogen and progesterone re‐infusion did not change heart rate responses to baroreceptor unloading (Brunt et al., 2013).

Aligning with our hypotheses, progesterone concentrations were negatively associated with cardiovagal BRS during the higher hormone phases. The negative association between progesterone and cardiovagal BRS during the higher hormone phase in all NAT and OCP women supports previous findings suggesting an antagonistic interaction between progesterone and oestrogen with BRS (Charkoudian, 2001; Hayashi et al., 2006). Specifically, oestrogen and progesterone might have opposing influences on BRS, and the relative levels of oestrogen and progesterone throughout the menstrual cycle might cause transient increases or decreases in cardiovagal BRS (Charkoudian, 2001). Taken together, previous and present data suggest that the nature of the relationship between oestrogen or progesterone concentrations and cardiovagal BRS is likely to be highly dependent on the relative concentrations of other endogenous ovarian sex hormones.

The regression model with ovarian sex hormone concentrations and group (OCP or NAT) revealed that group alone (independent of ovarian hormone concentrations) is significantly associated with cardiovagal BRS during the lower hormone phase. This suggests that inherent differences between NAT and OCP women (exogenous sex hormone fluctuations or types of progestins used in OCPs, for example) unrelated to circulating endogenous ovarian hormone concentrations influence BRS during the lower hormone phase. The regression model findings and the previous simple regression findings taken together suggest that relative levels of endogenous ovarian sex hormones and OCP generations, formulations or inflammatory profile all influence cardiovagal BRS. Furthermore, given that blood pressure is regulated by both cardiac output and total peripheral resistance, cardiovagal BRS (modulating changes in heart rate) is linearly associated with sympathetic BRS (modulating changes in total peripheral resistance) in women (Dutoit et al., 2010). Oestrogen attenuates sympathetically mediated changes in peripheral resistance, potentially via vascular β‐adrenergic receptors (Hart et al., 2011). Indeed, previous literature suggests greater sympathetic BRS during the higher hormone phase in comparison to the lower hormone phase in OCP women (Minson et al., 2000b), plausibly overcoming inhibited sympathetic transduction. Sympathetic transduction might play a role in the relationship between cardiovagal BRS and sex hormone fluctuations, because previous literature suggests a negative relationship between sympathetic BRS and sympathetic transduction in young, healthy men but not women (Hissen et al., 2019). However, cardiovagal and sympathetic BRS exhibit a moderate, linear association in young, healthy women (Dutoit et al., 2010). Perhaps low sympathetic BRS is compensated for by higher cardiovagal BRS in OCP women during the low hormone phase, as was noted in the present study. However, future studies are needed to test these relationships directly.

### 4.4 Limitations

The present study has some limitations. First, measurements of circulating endogenous ovarian hormone concentrations in OCP women do not take into account the circulating synthetic estradiol concentrations, and exogenous ethinyl‐estradiol and progestin in oral contraceptives suppress endogenous sex hormones. Second, the higher hormone phase includes ovulation and ≤4 days following ovulation, meaning that oestrogen and progesterone concentrations were either rising or falling at varying degrees in different participants. To reduce the impact of this variation, a relatively large sample size (*n* = 43) was used in the present study. Furthermore, oestrogen‐to‐progesterone ratio was not significantly associated with cardiovagal BRS (data not shown), supporting that the relative fluctuations of oestrogen and progesterone did not impact the present results. Third, non‐invasive, spontaneous measures of cardiovagal BRS were used, and thus, the present findings should be expanded upon by future studies incorporating lower‐body negative pressure, neck suction/pressure or the modified Oxford protocol to obtain a wider range of blood pressures for a true BRS curve, or by adding sympathetic BRS measurements.

## 5 CONCLUSION

In summary, cardiovagal BRS does not differ between the low and high hormone phases of the menstrual or pill cycle in NAT and OCP women, respectively. However, cardiovagal BRS is lower in NAT compared with OCP women during the early follicular/placebo phase of the menstrual/pill cycle, and lower cardiovagal BRS might be associated with higher endogenous oestrogen concentrations. In contrast, during the late follicular to early luteal/active pill phase, higher progesterone concentrations were predictive of lower cardiovagal BRS. The present study highlights the potential role of endogenous ovarian sex hormones and OCP generation or formulation on cardiovagal BRS in young, healthy women.

## AUTHOR CONTRIBUTIONS

Sara E. Mascone and Sushant M. Ranadive designed the research; Sara E. Mascone, Lauren E. Eagan, Dain W. Jacob and Jennifer L. Harper played a primary role in data collection; Sara E. Mascone processed and extracted BRS data files; Sara E. Mascone drafted figures; Sara E. Mascone, Sushant M. Ranadive, Dain W. Jacob and Jacqueline K. Limberg interpreted results; Sara E. Mascone, Sushant M. Ranadive, Dain W. Jacob and Jacqueline K. Limberg drafted the manuscript; all authors edited, revised, and approved the final version of the manuscript and agree to be accountable for all aspects of the work in ensuring that questions related to the accuracy or integrity of any part of the work are appropriately investigated and resolved. All persons designated as authors qualify for authorship, and all those who qualify for authorship are listed.

## CONFLICT OF INTEREST

All authors declare that they have no conflicts of interest.

## Supporting information


**Supplementary Figure S1**. Univariate linear regressions of oestrogen concentrations (left graph) or progesterone concentrations (right graph) and mean gain for the low and high hormone phases combined. Significant relationships are denoted by a continuous line and bolded *R* ^2^ and *P*‐values. Abbreviations: E2, oestrogen; P4, progesterone.

## Data Availability

The data that support the findings of this study are available from the corresponding author upon reasonable request.
