# Supplementary material for: Naturally menstruating women exhibit lower cardiovagal baroreflex sensitivity than oral contraceptive users during the lower hormone phase
Source: Exp Physiol. 2023 Oct 25;108(12):1481–9. doi: 10.1113/EP091394 (PMC10843041; doi:10.1113/EP091394)
Supplement: Supplementary file 1 — Supplementary Figure S1. Univariate linear regressions of oestrogen concentrations (left graph) or progesterone concentrations (right graph) and mean gain for the low and high hormone phases combined. Significant relationships are denoted by a continuous line and bolded R 2 and P‐values. Abbreviations: E2, oestrogen; P4, progesterone. [file EPH-108-1481-s001.docx]

**Supplementary Figure**

**Supplementary Figure 1.** Univariate linear regressions of estrogen concentrations (left graph) or progesterone concentrations (right graph) and Mean Gain for the low and high hormone phases combined. Significant relationships are denoted by a solid line and bolded R^2^ and p values. E2, estrogen; P4, progesterone.
